# Essential Role of Histone Replacement and Modifications in Male Fertility

**DOI:** 10.3389/fgene.2019.00962

**Published:** 2019-10-08

**Authors:** Tong Wang, Hui Gao, Wei Li, Chao Liu

**Affiliations:** ^1^State Key Laboratory of Stem Cell and Reproductive Biology, Institute of Zoology, Chinese Academy of Sciences, Beijing, China; ^2^College of Life Sciences, University of Chinese Academy of Sciences, Beijing, China

**Keywords:** spermiogenesis, histone-to-protamine transition, histone variants, histone modification, male infertility

## Abstract

Spermiogenesis is a complex cellular differentiation process that the germ cells undergo a distinct morphological change, and the protamines replace the core histones to facilitate chromatin compaction in the sperm head. Recent studies show the essential roles of epigenetic events during the histone-to-protamine transition. Defects in either the replacement or the modification of histones might cause male infertility with azoospermia, oligospermia or teratozoospermia. Here, we summarize recent advances in our knowledge of how epigenetic regulators, such as histone variants, histone modification and their related chromatin remodelers, facilitate the histone-to-protamine transition during spermiogenesis. Understanding the molecular mechanism underlying the modification and replacement of histones during spermiogenesis will enable the identification of epigenetic biomarkers of male infertility, and shed light on potential therapies for these patients in the future.

## Introduction

Spermatogenesis is the process of male gamete production with successive cellular differentiation, which can be subdivided into spermatogonial mitosis, spermatocytic meiosis and spermiogenesis (Roosen-Runge, 1962; Hess and Renato De Franca, 2008). During spermatogenesis, SSC (spermatogonial stem cells) undergo self-renewal and differentiate into spermatogonia that perform meiosis to generate haploid germ cells and ensure the genetic diversity through meiotic recombination (Rathke et al., 2014; Bao and Bedford, 2016). Then, the haploid germ cells undergo spermiogenesis with a distinct morphological change and chromatin compaction in the sperm nuclei to prevent the paternal genome from mutagenesis and damage (Govin et al., 2004; Bao and Bedford, 2016). During the nuclear chromatin re-organization in spermiogenesis, the majority of the somatic histones are firstly replaced by testis-specific histone variants, and transition proteins (TPs) are subsequently incorporated in the nuclei of spermatids, protamines (PRMs) further replace TPs in the late spermatids to pack the genome into the highly condensed sperm nucleus (Rathke et al., 2014; Bao and Bedford, 2016). During the histone-to-protamine transition, the histone variants and specific histone modifications play essential roles by modulating the chromatin compaction and higher-order chromatin structure (**Table 1**) (Boskovic and Torres-Padilla, 2013; Bao and Bedford, 2016; Hada et al., 2017; Hao et al., 2019). Defects in either the replacement or the modification of histones might result in azoospermia, oligospermia or teratozoospermia, which leads to male infertility (**Table 2**). The focus of this review is on recent advances in our knowledge of how epigenetic regulators, such as histone variants, histone modification and their related chromatin remodelers, regulate the highly orchestrated chromatin re-organization and facilitate the histone-to-protamine transition during spermiogenesis.

**Table 1:** The main histone variants and modifications during the histone-to-protamine transition.

Type	Histone	Name	Period	Function	Reference
Histone variants	H1	H1T	Spermatocytes to elongating spermatids	Maintain open chromatin configuration and no detectable phenotype in H1t-null testis	Delucia et al., 1994; Khadake and Rao, 1995; Drabent et al., 2000; Fantz et al., 2001
		H1T2	Round spermatids and elongating spermatids	Indispensable for the replacement of histones with protamines and chromatin condensation	Martianov et al., 2005; Tanaka et al., 2005
		HILS1	Elongating and elongated spermatids	Contribute to the open chromatin structure	Yan et al., 2003; Mishra et al., 2018
	H2A	TH2A	Spermatocytes to elongated spermatids	Contribute to the open chromatin structure and cooperate with TH2B to regulate TP2 incorporation	Padavattan et al., 2015; Shinagawa et al., 2015; Padavattan et al., 2017
		H2AL2	Elongating and elongated spermatids	Assemble open nucleosomes and allow TPs incorporation	Govin et al., 2007; Barral et al., 2017
		H2A.B	Spermatocytes to round spermatids	Destabilize chromatin and modulate the dynamics of H2AL2 removal and TP1 incorporation and	Soboleva et al., 2012; Soboleva et al., 2017; Anuar et al., 2019
	H2B	Th2B	Spermatocytes, round spermatids and elongating spermatids	Destabilize chromatin and regulate the TPs and PRMs incorporation	Meistrich et al., 1985; Montellier et al., 2013
	H3	H3.3	All types of germ cell	Contribute to the open chromatin structure, modulate TP1 removal and PRM1 incorporation	Bramlage et al., 1997; Couldrey et al., 1999; Van Der Heijden et al., 2007; Thakar et al., 2009; Chen et al., 2013; Tang et al., 2015
		H3T	Spermatocytes, round spermatids and elongating spermatids	Contribute to the open chromatin and required for spermatogonial differentiation and ensures entry into meiosis	Tachiwana et al., 2010; Ueda et al., 2017
Histone modifications	Acetylation	H4K5/8/12ac	Spermatogonia, spermatocytes and elongating spermatids	Essential for destabilization and remodeling of nucleosomes, TPs incorporation	Hazzouri et al., 2000;Gaucher et al., 2012; Qian et al., 2013; Bell et al., 2014; Dong et al., 2017; Ketchum et al., 2018
		H4K16ac	Elongating spermatids		
	Ubiquitination	UbH2A	Spermatocytes and elongating spermatids	Essential for the recruitment of the MOF acetyltransferase complex to modulate H4K16ac and histone removal	Chen et al., 1998; Baarends et al., 1999; Lu et al., 2010; Gou et al., 2017; Meng et al., 2019; Wang et al., 2019
		UbH2B	Spermatocytes and elongating spermatids		
	Methylation	H3K4me3	Spermatogonia, spermatocytes, round spermatids and elongating spermatids	Essential for the recruitment of PYGO2 to recognize HAT to facilitate H3 acetylation; recruit PHF7 to catalyze H2A ubiquitination to facilitate the histone removal	Godmann et al., 2007; Song et al., 2011; Nair et al., 2008; Wang et al., 2019
		H3K9me1/2/3	Spermatogonia, Round spermatids and elongating spermatids	Regulate the *Tnps* and *Prms* genes expression	Okada et al., 2007
		H3K36me3	Spermatocytes and round spermatids	Regulate the *Tnps* and *Prms* genes expression	Zuo et al., 2018
		H3K79me3	Elongating spermatids	Correlate with histone H4 hyperacetylation to regulate histone-to-protamine transition	Dottermusch-Heidel et al., 2014
	Phosphorylation	γH2AX	Spermatocytes elongating spermatids	Require for the normal quantities of H3, H4 and PRM2 precursor and intermediate	Li et al., 2005; Spiridonov et al., 2005; Jha et al., 2017
		H4S1	Spermatocyte, round spermatids and elongating spermatids	Essential for chromatin compaction and concomitantly histone accessibility	Krishnamoorthy et al., 2006; Zhang et al., 2016
	Other	Crotonylation	Elongating spermatids	Facilitate TP1 and PRM2 incorporation	Liu et al., 2017b
		PARsylation	Elongating spermatids	Require for histone removal and TP1 incorporation	Meyer-Ficca et al., 2009; Meyer-Ficca et al., 2011; Meyer-Ficca et al., 2015

**Table 2:** Mouse models related with the histone-to-protamine transition.

Gene	Phenotype	Function	Reference
*H1t*	Fertility and no spermatogenesis abnormalities	Dispensable for histone-to-protamine transition	Drabent et al., 2000; Fantz et al., 2001
*H1t2*	Reduced fertility with delayed nuclear condensation and aberrant elongation of spermatids	Indispensable for the incorporation of PRMs and proper chromatin condensation	Martianov et al., 2005; Tanaka et al., 2005
*Th2b*	Fertility with normal spermatogenesis in *Th2b*-null mice	Destabilize chromatin and regulate TPs and PRMs incorporation	Montellier et al., 2013
	Infertility with abnormal spermatozoa in TH2B C-terminus modified mice		
*Th2a/Th2b*	Infertility with accumulated spermatocytes at interkinesis and abnormal spermatozoa	Indispensable for cohesin release and TP2 incorporation	Shinagawa et al., 2015
*H2al2*	Infertility and the sperm chromatin show a compaction defects	Assemble open nucleosomes and allow TPs incorporation	Barral et al., 2017
*H2a.b*	Reduced fertility with abnormal spermatozoa	Destabilize chromatin and modulate the dynamics of H2AL2 removal and TP1 incorporation	Anuar et al., 2019
*H3f3a*	Reduced fertility with dysmorphic spermatozoa	Require for normal development of some spermatids	Tang et al., 2015
*H3f3b*	Infertility with abnormal spermatozoa and reduced sperm count	Indispensable for spermatogenesis related genes expression, TP1 removal and PRM1 incorporation	Yuen et al., 2014
*H3t*	Infertility with azoospermia	Require for spermatogonial differentiation and ensures entry into meiosis	Ueda et al., 2017
*Epc1*	Infertility with abnormal round spermatids to elongating spermatids transition	Require for round spermatids maturation by regulating histone acetylation and TP2 incorporation	Dong et al., 2017
*Tip60*	The germ cell is arrested at the RS stage	Contribute to round spermatids maturation by regulating histone acetylation and TP2 incorporation	Dong et al., 2017
*Sirt1*	Reduced fertility with abnormal spermatozoa and decreased sperm count	Require for acetylation of H4K5, H4K8 and H4K12 histone, and TP2 incorporation	Bell et al., 2014
*Brdt*	Infertility with complete absence of post-meiotic cells in *Brdt*-null mice	Control the chromatin organization and meiotic sex chromosome inactivation; the first bromodomain of BRDT is essential to link histone removal and TPs, PRMs incorporation	Shang et al., 2007; Dhar et al., 2012; Gaucher et al., 2012; Manterola et al., 2018
	Infertility with abnormal spermatids in *Brdt* *^ΔBD1/ΔBD1^* mice		
*Pa200*	Reduced fertility with abnormal spermatozoa and decreased sperm count	Recognize acetylated histones and mediate the core histones for acetylation dependent degradation through proteasomes	Khor et al., 2006; Qian et al., 2013
*Rnf8*	Infertility with abnormal spermatozoa and reduced sperm count	Require for histone ubiquitination and modulate H4K16ac to facilitate histone removal and TPs, PRMs incorporation	Lu et al., 2010
*Miwi*	Infertility with abnormal spermatozoa and reduced sperm count in *Miwi* D-box mutations mice	Essential for nuclear translocation of RNF8 and facilitates the histone ubiquitination and further histone removal	Gou et al., 2017
*L3mbtl2*	Reduced fertility with abnormal spermatozoa and decreased sperm count	Require for the RNF8-UbH2A pathway and further PRM1 incorporation	Meng et al., 2019
*Phf7*	Infertility with abnormal spermatozoa and decreased sperm count	Recognize the H3K4me3/me2 and catalyze H2A ubiquitination to facilitate the histone removal	Wang et al., 2019
*Pygo2*	Infertile with abnormal spermatozoa and decreased sperm count in *Pygo2* reduced mice	Recognize H3K4me3 and recruit HAT to facilitate H3 acetylation and expression of *Prms*, *Tnp2*, and *H1fnt*.	Nair et al., 2008
*Jhdm2a*	Infertile with abnormal spermatozoa and decreased sperm count	Control H3K9 methylation at the promoter of *Tnp1* and *Prm1* genes and regulate their expression	Okada et al., 2007
*Setd2*	Infertility with round spermatid arrest	Catalyze H3K36me3 and facilitate the activation of *Tnp*s and *Prms* genes	Zuo et al., 2018
*Tssk6*	Infertility with abnormal spermatozoa	Mediate γH2AX to possess normal quantities of histone H3, H4 and PRM2 precursor and intermediate	Spiridonov et al., 2005; Jha et al., 2017
*Cdyl*	Reduced fertility with decreased sperm count and motility	Regulate histone crotonylation to facilitate TP1 and PRM2 incorporation	Liu et al., 2017b
*Parp11*	Infertility with teratozoospermia	Modulate PARsylation to facilitate chromatin condensation	Meyer-Ficca et al., 2015
*Parg110*	Reduced fertility with poor sperm chromatin quality	Dispensable for histone removal and TP1 incorporation	Meyer-Ficca et al., 2009; Meyer-Ficca et al., 2011
*Tnp1*	Reduced fertility with subtle abnormal spermatozoa and decreased spermatozoa	Dispensable for histone displacement as the compensation by TP2 and PRM2 precursor	Yu et al., 2000
*Tnp2*	Reduced fertility with abnormal spermatozoa	Dispensable for histone displacement but necessary for maintaining the normal processing of PRM2 and the completion of chromatin condensation	Zhao et al., 2001
*Tnp1/Tnp2*	Infertile with abnormal spermatozoa and decreased sperm count	Indispensable for PRM2 incorporation and chromatin condensation	Shirley et al., 2004
*Prm1*	Infertile with abnormal spermatozoa and decreased sperm count	Indispensable for spermiogenesis and chromatin condensation	Cho et al., 2001
*Prm2*	Infertile with abnormal spermatozoa and decreased sperm count		
*Camk4*	Infertile with abnormal spermatozoa and decreased sperm count	Mediate the phosphorylation of PRM2 and facilitate basic nuclear proteins removal	Wu et al., 2000

## Histone Variants

In eukaryotes, nucleosomes are the packing units of DNA, which contain four types of canonical histones (H2A, H2B, H3, and H4) and the linker histone H1 (Talbert and Henikoff, 2010; Kowalski and Palyga, 2012). While canonical histone expression is typically coupled to DNA replication, some non-canonical histones (histone variants) that are distinct form their canonical paralogues in amino acid sequence, are constitutively expressed and have roles in a wide range of processes (Talbert and Henikoff, 2010). Many histones variants are expressed during spermiogenesis and modulate the chromatin structure to facilitate the histone-to-protamine replacement (Mccarrey et al., 2005; Govin et al., 2007). Here, we summarize the recent advances in our understanding of the role of histone variants during the histone-to-protamine transition.

## H1 Variants

**Figure 1:**
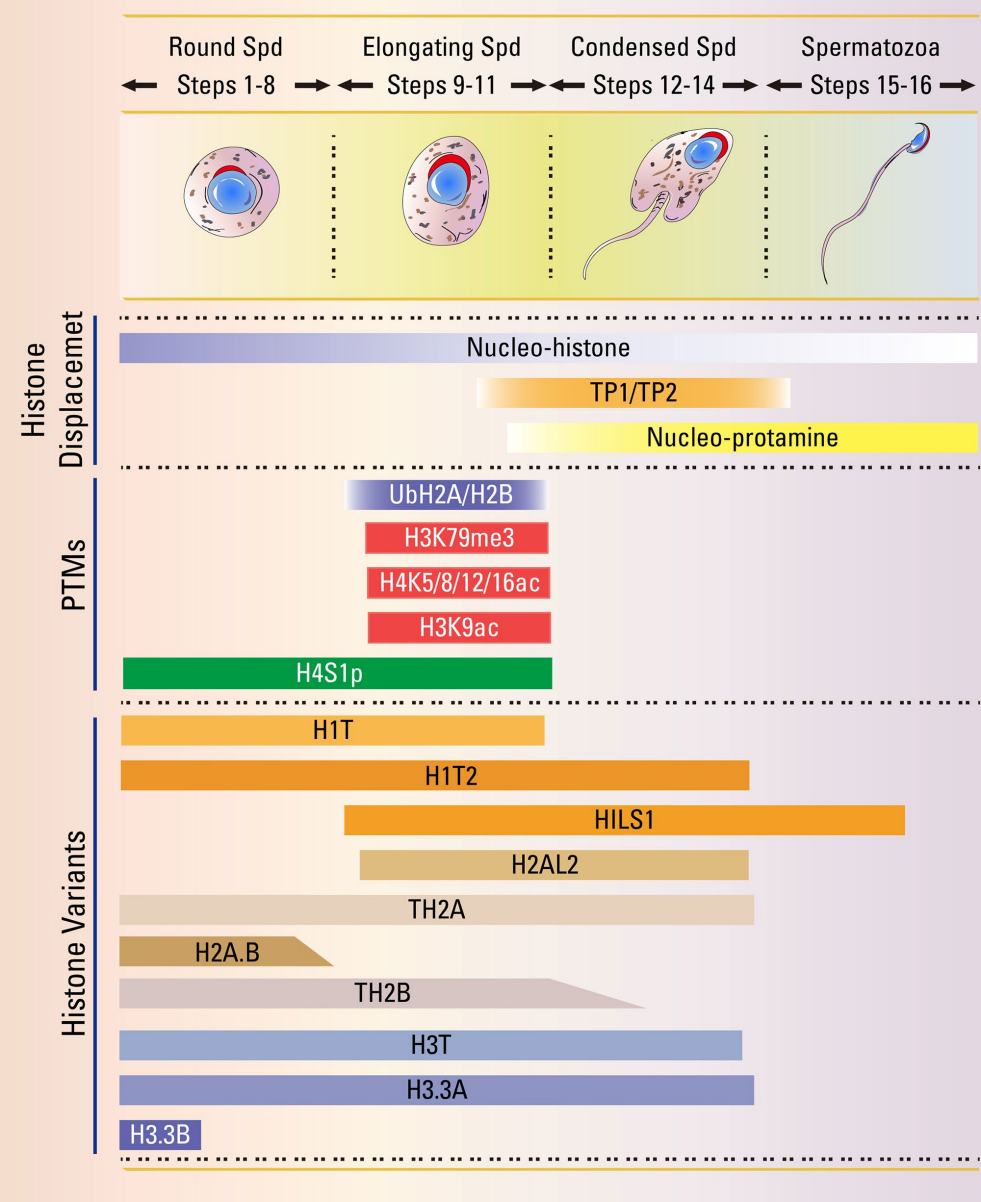
Summary of critical time points and epigenetic events during the histone-to-protamine transition. The haploid germ cells undergo a dramatic morphological change, and nuclear chromatin re-organization starts from round spermatid (Spd) to mature spermatozoa. Detailed studies of the indicated histone variants and histone modification might establish the precise epigenetic events of spermiogenesis.

Linker histones contribute to form and stabilize the higher-order chromatin structure (Bednar et al., 1998). In mammals, there are about 11 different subtypes of histone H1 (Happel and Doenecke, 2009). Among these, H1T, H1T2, and HILS1 are testis-specific H1 variants (**Figure 1**) (Happel and Doenecke, 2009).

H1T is exclusively detected as early as mid- to late pachytene spermatocytes, and maintains high expression levels in the elongating spermatids (**Figure 1**) (Drabent et al., 1996; Drabent et al., 2003). Biochemical and biophysical studies found that, distinct from other somatic H1 variants, H1T binds less tightly to H1-depleted nucleosomes, suggesting it may maintain a relatively open chromatin configuration to facilitate histone replacement during spermiogenesis (Delucia et al., 1994; Khadake and Rao, 1995). Unexpectedly, *H1t*-null mice are fertile and exhibit no spermatogenesis abnormalities, and the histone-to-protamine transition in *H1t*-deficient testis is normal (Drabent et al., 2000; Fantz et al., 2001). Although the expression of some canonical subtypes, including H1.1, H1.2, and H1.4, is enhanced in *H1t*-null mice, elevated levels of H1.1 or H1.2 could not be observed in the *H1t*-deficient spermatids (Drabent et al., 2003), indicating some other types of H1 variants may play redundant roles in the histone-to-protamine transition.

H1T2 selectively localizes at the apical pole in the nucleus of round and elongating spermatids but not in mature spermatozoa (**Figure 1**) (Martianov et al., 2005). Distinct from H1T, H1T2 is critical for spermiogenesis, as homozygous *H1t2*-mutant males are infertile due to delayed nuclear condensation and aberrant elongation of spermatids. Further analysis shows the protamine levels are substantially reduced in *H1t2*-null spermatozoa (Martianov et al., 2005; Tanaka et al., 2005), indicating H1T2 is necessary for the incorporation of protamines, and proper chromitin condensation during the histone-to-protamine transition.

HILS1 is strongly expressed in the nuclei of elongating and elongated spermatids (**Figure 1**) (Yan et al., 2003). HILS1 is the least conserved H1 variant, and a poor condenser of chromatin compared with somatic H1, demonstrating the idea that HILS1 may have a distinct role in the histone-to-protamine transition (Yan et al., 2003; Mishra et al., 2018). In *Drosophila*, *Mst77F* encodes a linker histone-like protein that is similar with the mammalian HILS1 protein and expressed in elongating spermatids (Raja and Renkawitz-Pohl, 2005). The disruption of *Mst77F* cause male sterile as producing spermatozoa with malformed heads. Although the histone-to-protamine transition occurs independently of *Mst77F*, the nuclei of spermatid fail to properly condense after the histone-to-protamine replacement in *Mst77F* mutant male (Kimura and Loppin, 2016). However, the functional roles of HILS1 in mammalian spermiogenesis need further investigation.

## H2a Variants

Multiple testis-specific H2A variants have been identified in mammals, including TH2A, H2AL1, H2AL2, H2AL3 and H2A.B (Trostleweige et al., 1982; Govin et al., 2007; Soboleva et al., 2012).

TH2A is present and actively synthesized in early primary spermatocytes and gradually disappears during condensation of spermatid nuclei (**Figure 1**) (Shires et al., 1976; Trostleweige et al., 1982). TH2A could contribute to the open chromatin structure, as crystal structures of nucleosome core particles (NCPs) with TH2A show the H-bonding interactions between the TH2A/TH2A′ L1 loops are lost and the histone dimer-DNA contacts are dramatically decreased (Padavattan et al., 2015; Padavattan et al., 2017). Although a *Th2a*-knockout mouse model has yet to be established, mice with knockouts of the testis-specific H2B variants *Th2a* and *Th2b* exhibit male infertility with few sperm in the epididymis (Shinagawa et al., 2015). In this double-knockout mouse, impaired chromatin incorporation of transition protein 2 (TP2) and elevated H2B could be observed in the mutant testis, suggesting the TH2A and TH2B may regulate the function in chromatin dynamics or the total histone levels to facilitate the histone replacement during spermatogenesis (Shinagawa et al., 2015). As the *Th2b*-null male mice show normal spermatogenesis and fertility (Montellier et al., 2013), the histone replacement defect in *Th2a*/*Th2b* double-knockout male mice is probably caused by the depletion of *Th2a* or their synergistic effect.

In late-developing post-meiotic male germ cells, H2AL2 is specifically expressed in condensing spermatids that correlates with the expression of TPs (**Figure 1**) (Govin et al., 2007). By comparing *H2al2*-null mice to wild-type mice, H2AL2 was demonstrated to be required to load TPs onto the nucleosome and for efficient PRMs assembly during the histone-to-protamine transition. Additionally, the nucleosome reconstitution assays revealed that the incorporation of H2A.L.2 can drastically modulate the nucleosome structure to facilitate TPs invading the nucleosomes and further transformation (Barral et al., 2017). Thus, H2AL2 could assemble open nucleosomes and allow TPs invading, which further promotes protamine processing and sperm genome compaction.

H2A.B is spatially and temporally regulated during spermatogenesis and detectable from the pachytene stage to the round spermatids (**Figure 1**) (Soboleva et al., 2012; Soboleva et al., 2017). *In vitro* studies show that H2A.B is able to destabilize chromatin and has unfolding properties to chromatin (Soboleva et al., 2012), indicating H2A.B might promote chromatin reorganization and further histones displacement by TPs. Male *H2a.b*-null male mice are subfertile due to the production of abnormal spermatozoa and clogged seminiferous tubules (Anuar et al., 2019). In *H2a.b*-null elongating spermatids, H2AL2 could not be detected in pericentric heterochromatin, and the replacement of TP1 by protamines appears to be delayed (Anuar et al., 2019). These results indicate H2A.B might modulate the dynamics of H2AL2 and TP1 chromatin incorporation and removal to participate in the histone-to-protamine transition.

## H2b Variants

The testis-specific histone variant TH2B is one of the earliest histone variants identified in testis (Shires et al., 1975). TH2B massively replaces somatic H2B during meiosis and remains the main type of H2B in round and elongating spermatids (Meistrich et al., 1985; Montellier et al., 2013), suggesting TH2B might be indispensable for meiotic and post-meiotic germ cells. The crystal structure analysis shows the TH2B could not form the water-mediated hydrogen bonds with H4R78 (Urahama et al., 2014), which may affect the stability of the TH2B nucleosome and facilitate histone replacement during spermiogenesis. In a *Th2b* mutant mouse, which contains modified C-terminus of the TH2B protein and causes a dominant-negative effect, males were infertile and severe abnormalities were seen in the elongating spermatids, which affected subnucleosomal transitional states during histone replacement (Boskovic and Torres-Padilla, 2013; Montellier et al., 2013). In contrast, *Th2b*-null mice are fertile and show normal spermatogenesis process, indicating a compensatory mechanism that rescues deficiency of TH2B in the histone-to-protamine transition. Indeed, in *Th2b*-null testis, the expression of somatic H2B was significantly increased and elevated methylation of H4R35, H4R55, H4R67, and H2BR72 could be detected in *Th2b*-null spermatids. As H4R35, H4R55, H4R67, and H2BR72 participate in the interactions of histone–DNA and histone–histone, and their methylation may impair these intranucleosomal interactions (Hoghoughi et al., 2018). Thus, the elevated somatic H2B and histone modification in *Th2b*-null spermatids might rescue the *Th2b* deficiency in testis (Montellier et al., 2013; Bao and Bedford, 2016).

In humans, H2BFWT is a testis-specific histone, is synthesized and aggregated in testes, and single nucleotide polymorphisms (SNPs) in this gene is highly associated with male infertility (Churikov et al., 2004; Lee et al., 2009; Ying et al., 2012; Rafatmanesh et al., 2018; Teimouri et al., 2018). And spermatid-specific H2B (ssH2B) and H2BL1 have been identified and are strongly enriched in round or elongating spermatids, similar to that of TPs and protamines (Moss and Orth, 1993; Unni et al., 1995; Govin et al., 2007). However, the functional roles of these H2B variants in the histone-to-protamine transition still need to be further elucidated.

## H3 Variants

In addition to the two canonical histones H3.1 and H3.2, three additional H3 variants have been identified and expressed in mammal testes, including H3.3, H3T and H3.5 (Rathke et al., 2014; Bao and Bedford, 2016).

H3.3 differs from canonical H3.1 with five amino acids, is expressed throughout mouse seminiferous tubules, and accumulates in the XY body of spermatocytes (Bramlage et al., 1997; Van Der Heijden et al., 2007). Biochemical and biophysical studies show that H3.3 contributes to an open chromatin configuration and promotes transcription through disrupting the higher-order chromatin structure (Thakar et al., 2009; Chen et al., 2013). H3.3 could be encoded by two gene paralogs in mammal, *H3f3a* and *H3f3b*, and the depletion of either *H3f3a* or *H3f3b* causes male infertility. The disruption of *H3f3a* produces abnormal spermatozoa (Couldrey et al., 1999; Tang et al., 2015), and the loss of *H3f3b* leads to growth defects and death at birth, with surviving *H3f3b*-null males showing complete infertility (Yuen et al., 2014). In *H3f3b*-null germ cells, the TP1 is abnormally deposited in elongating spermatids while PRM1 could not be observed in in elongated spermatids and mature spermatozoa, indicating that *H3f3b* is required for chromatin reorganization and the histone-to-protamine transition (Yuen et al., 2014). H3T (H3.4) is exclusively expressed in the spermatocyte and diminishes in the elongating spermatids (Ueda et al., 2017). Biochemical studies clearly indicate that, in the H3T nucleosome, the DNA around the entry-exit regions shows more flexible than that of the H3.1-containing nucleosome, and that the H3T-containing polynucleosome could formed more open configuration than that of H3.1 (Tachiwana et al., 2010). However, the disruption of H3T leads to sterile males with azoospermia, as spermatocyte and spermatids are absent in the *H3t*-null testes (Ueda et al., 2017). Thus, the function of H3T in the later stage of spermatogenesis need further investigated by using spatially and temporally specific knockout mouse models.

H3.5 is highly expressed in human testis and specifically observed in spermatogonia and spermatocytes (Shiraishi et al., 2017). *In vitro* studies reveal that the H3.5-specific L103 residue, reduces the hydrophobic interaction with histone H4 in the H3.5-containing nucleosome, which corresponds to the H3.3 Phe104 residue (Urahama et al., 2016). H3.5 is significantly reduced in non-obstructive azoospermia (NOA) patients (Shiraishi et al., 2017), whereas the precise roles of H3.5 in spermatogenesis remain largely unknown.

## Histone Modification

Covalent conjugation of different post-translational modification of histones has a dramatic effect on the chromatin conformation by affecting the stability of the nucleosome and the histone-DNA interaction (Bao and Bedford, 2016). Many types of histone modifications have been identified to facilitate the histone-to-protamine transition, including acetylation, ubiquitination, methylation, and phosphorylation (Luense et al., 2016).

## Acetylation

Hyperacetylated histones could facilitate histone eviction, and the acetylation of H2A, H2B, H3, H4 and histone variants have been detected in mammal testis (Grimes and Henderson, 1984a; Grimes and Henderson, 1984b; Oliva and Mezquita, 1986; Oliva et al., 1987). In *Drosophila*, inactivation of histone acetyltransferases by anacardic acid prevents the histones degradation and further a protamine incorporation during spermiogenesis (Awe and Renkawitz-Pohl, 2010), suggesting that histone acetylation is essential for the histone-to-protamine replacement.

**Figure 2:**
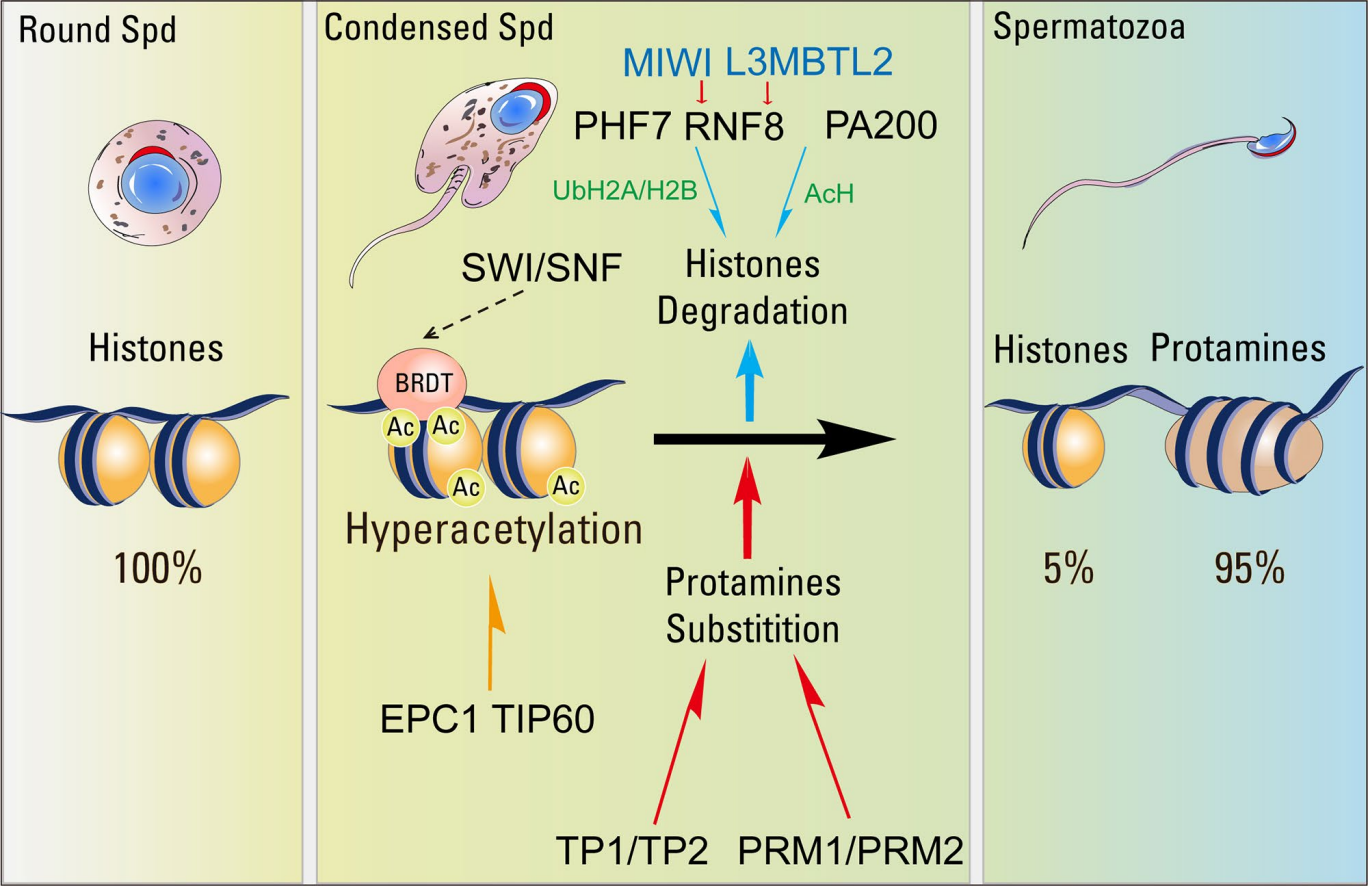
The key factors related to the histone-to-protamine transition. Global incorporation of various H2A, H2B and H3 histone variants creates highly unstable nucleosomes, which then undergo histone hyperacetylation by EPC1/TIP60 or some other nucleosome acetyltransferase complexes. Acetylation at critical lysines further destabilizes the nucleosomes, while tail acetylation generates a platform for the recruitment of BRDT. BRDT interacts with the SWI/SNF family protein then starts the process of histone eviction and replacement by TPs. Evicted acetylated histones would then be recognized by PA200 and degraded by proteasomes during spermatogenesis. RNF8 could catalyze the ubiquitination of H2A. Ubiquitinated H2A and H2B control H4K16ac by regulating the association of MOF to the chromatin and facilitates histone removal in elongating spermatids. MIWI binds to RNF8 in the cytoplasm of early spermatids (Spd) through a Piwi-interacting RNA (piRNA)-independent manner, and promotes the nuclear translocation of RNF8 in late spermatids to catalyze histone ubiquitination and trigger histone removal. L3MBTL2 could interact with RNF8 and facilitate RNF8-dependent histone ubiquitination-related histone removal. PHF7 could recognize the H3K4me3/me2 and catalyze H2A ubiquitination to facilitate histone removal in elongating spermatids.

H4 acetylation (H4K5ac, H4K8ac, H4K12ac, and H4K16ac) shows a spatial distribution pattern during spermatogenesis and is indispensable for the histone-to-protamine transition (Bao and Bedford, 2016; Ketchum et al., 2018). H4K5ac, H4K8ac and H4K12ac are expressed in spermatogonia and pre-leptotene spermatocytes, disappear in leptotene to pachytene spermatocytes, reappeared in elongating spermatids, and finally disappeared in condensing spermatids (**Figure 1**) (Hazzouri et al., 2000; Ketchum et al., 2018). In contrast, H4K16ac could only be detected in elongating spermatids (**Figure 1**) (Ketchum et al., 2018). *In vitro* analysis shows that H4 acetylation is essential for destabilization and remodeling of nucleosomes, and the incorporation of H4K16ac into nucleosomes prevents the formation of compact chromatin fibers and influence chromatin forming cross-fiber interactions (Tse et al., 1998; Shogren-Knaak et al., 2006; Kan et al., 2009). These findings indicate that H4 acetylation modulates higher order chromatin structure to facilitate the histone-to-protamine transition. EPC1 (Enhancer Of Polycomb Homolog 1) and TIP60 (Tat-interactive protein, 60 kDa), which are two components for the mammalian NuA4 (nucleosome acetyltransferase of H4) complexes (**Figure 2**) (Doyon et al., 2004), are co-localized to the nuclear periphery near the acrosomes in both round spermatids and elongating spermatids (Dong et al., 2017). The depletion of either *Epc1* or *Tip60* perturbs histone hyperacetylation, especially H4 acetylation, and affects histone replacement during spermiogenesis (Dong et al., 2017). Another gene that may play a role in acetylation is SIRT1 (Sirtuin 1), a member of the NAD+-dependent deacetylase. Germ cell-specific *Sirt1* knockout mice display reduced male fertility due to decreased spermatozoa number and increased proportion of abnormal spermatozoa (Bell et al., 2014; Liu et al., 2017a). In *Sirt1*-null elongating and elongated spermatids, acetylation levels of H4K5, H4K8 and H4K12 are decreased and TP2 could not co-localize in the nucleus, leading to a chromatin condensation defect in *Sirt1*-null spermatozoa (Bell et al., 2014). Thus, SIRT1 may modulate other factors to promote H4 acetylation and the histone-to-protamine transition.

The histone acetylation might be recognized by some chromatin remodelers to confer downstream signaling, and the double bromodomain and extra-terminal domain (BET) proteins have been identified to be critical epigenetic readers binding to acetylated histones and modulating changes in chromatin structure and organization during spermiogenesis (Berkovits and Wolgemuth, 2013). BRDT is a testis-specific BET member protein, which is expressed specifically in spermatocytes and spermatids, and contains two bromodomains that specifically recognize acetylated lysine residues (Shang et al., 2007; Dhar et al., 2012; Manterola et al., 2018). BRDT binds the hyperacetylated histone H4 tail and co-localizes with acetylated H4 in elongating spermatids (Pivot-Pajot et al., 2003; Govin et al., 2006). Remodeling assays have shown BRDT regulated the chromatin reorganization dependent acetylation in round spermatids (Dhar et al., 2012). In mice, the disruption of the first bromodomain in BRDT resulted in male sterility by producing the morphologically abnormal spermatids (Shang et al., 2007). In elongating spermatids with BRDT containing a knockout of bromodomain 1 (BD1), TPs and protamines remained in the cytoplasm and histone replacement did not occur, suggesting BRDT is required for the histone-to-protamine transition by mediating the replacement of acetylated histones (**Figure 2**) (Gaucher et al., 2012). Furthermore, BRDT was found to bind with the N-terminus of SMARCE1 (SWI/SNF-related matrix-associated actin-dependent regulator of chromatin subfamily E member 1), a member of the SWI/SNF family of ATP-dependent chromatin remodeling complexes (Dhar et al., 2012), indicating BRDT may cooperate with SMARCE1 to facilitate the histone-to-protamine transition during spermiogenesis (**Figure 2**).

Proteasomes catalyze ATP- and polyubiquitin-dependent protein degradation, and they are made up of a 20S catalytic core particle (CP) and regulatory particle (RP). The 20S CP could be activated by cooperation with various RPs, such as PA700/19S, PA28α/β, PA28γ, and PA200 (Stadtmueller and Hill, 2011). PA200 is highly expressed in the testis, and the disruption of PA200 results in male infertility and severe defects in spermatogenesis (Ustrell et al., 2005; Khor et al., 2006). During spermiogenesis, PA200 regulatory could directly recognize acetylated histones through a bromodomain-like module and promote their ubiquitin-independent degradation. In *Pa200*-null spermatids, results showed that H2B, H3 and elevated H4K16ac could be detected at the end of the elongation stage (Qian et al., 2013). Thus, PA200 specifically recognizes acetylated histones and mediates the core histones for acetylation dependent degradation through proteasomes during spermatogenesis (**Figure 2**).

## Ubiquitination

Ubiquitin is a 76 amino acid protein that is attached to target proteins to regulate several cellular processes, such as protein degradation, cell signaling, autophagy, DNA damage responses and so on (Hershko and Ciechanover, 1998; Pickart, 2001; Welchman et al., 2005; Komander and Rape, 2012). Ubiquitinated H2A and H2B are enriched in spermatocytes and elongating spermatids (Chen et al., 1998; Baarends et al., 1999). RNF8 is an ubiquitin E3 ligase that participates in DDR (DNA damage repair) by catalyzing the ubiquitination of H2A to promote the recruitment of some DNA damage response factors on the damage sites (Ma et al., 2011). The disruption of *Rnf8* causes significant late-stage developmental defects in spermatids due to problematic histone-to-protamine replacement, with the canonical histones being detectable in *Rnf8*-deficient mature spermatozoa (Lu et al., 2010). In *Rnf8*-null mice, both ubiquitinated H2A and H2B are decreased in the testes and H4K16ac is dramatically decreased as well (Lu et al., 2010). Further studies showed that ubiquitinated H2A and H2B were essential for the efficient recruitment of the MOF (males absent on the first) acetyltransferase complex, which is highly expressed in elongating spermatids and responsible for H4K16 acetylation in the chromatin (Akhtar and Becker, 2000; Lu et al., 2010). Thus, RNF8 catalyzed histone ubiquitination could modulate H4K16ac by regulating the localization of MOF on the chromatin and facilitate histone removal in the elongating spermatids.

The RNF8-dependent histone ubiquitination during spermiogenesis could also be modulated by PIWI protein, which is specifically expressed during germline development and enlists piRNAs (Piwi-interacting RNAs) to repress TE (transposable elements) and protect the germ cell genome integrity (Juliano et al., 2011; Siomi et al., 2011; Gou et al., 2017). In mice, *Miwi*, *Mili*, and *Miwi2*, the *Piwi* paralogs, have been identified in the testis and are required for male fertility (Deng and Lin, 2002; Kuramochi-Miyagawa et al., 2004; Carmell et al., 2007). During spermiogenesis, MIWI binds to RNF8 in the cytoplasm of early spermatids through a piRNAs-independent manner, and APC/C mediated MIWI degradation in late spermatids is essential for nuclear translocation of RNF8, which catalyzes histone ubiquitination and further facilitates histone removal (Gou et al., 2017). In both humans and mice, mutations in the conserved destruction box (D-box) of HIWI and MIWI proteins, which lead to their stabilization, cause male infertility due to impaired histone ubiquitination and histone-to-protamine transition (Gou et al., 2017). Except MIWI, L3MBTL2 (Lethal 3 malignant brain tumor like 2), a member of the MBT-domain proteins that is implicated in chromatin compaction, could also interact with RNF8. The depletion of *L3mbtl2* in germ cells affected male fertility by producing abnormal spermatozoa and the decrease of sperm counts. *L3mbtl2* deficiency also caused the reduction of in levels of the RNF8 and histone ubiquitination in elongating spermatids, which further influenced the PRM1 deposition and chromatin condensation during spermiogenesis (Meng et al., 2019).

PHF7 (PHD Finger Protein 7), which contains PHD (plant homeodomain) and RING finger domain, has been identified as a novel H2A ubiquitination E3 ligase in mouse testis (Hou et al., 2012; Wang et al., 2019). PHF7 is specifically located in the elongating spermatid nuclei, and the disruption of *Phf7* led to male mouse infertility as reduction of sperm count and the increased proportion of abnormal spermatozoa (Wang et al., 2019). PHF7 could recognize the H3K4me3/me2 through its PHD domain and catalyze H2A ubiquitination by its RING domain. In *Phf7*-null spermatids, the H2A ubiquitination was dramatically decreased that resulted in the histone retention and protamine replacement defect (**Figure 2**) (Wang et al., 2019). Therefore, PHF7 has dual roles during the histone-to-protamine transition that works as an epigenetic reader by recognizing H3K4me3/me2 and as an epigenetic writer through catalyzing H2A ubiquitination to promote histone removal.

## Methylation

Multiple histone methylation have been identified in elongating spermatids, for instance H3K4me2, H3K4me3, H3K9me2, H3K9me3, H3K27me3, H3K79me2, and H3K79me3 (Godmann et al., 2007; Song et al., 2011; De Vries et al., 2012; Dottermusch-Heidel et al., 2014). Among them, the methylation of H3K4 and plus acetylation might help to achieve a more-open chromatin configuration, whereas H3K9 and H3K27 methylation are known to be associated with a more-repressed chromatin configuration (Rathke et al., 2014), indicating a balance of “opened” and “closed” chromatin regions during the histone-to-protamine transition. As some histone methyltransferases and demethylases are detectable during spermiogenesis (Godmann et al., 2007; Liu et al., 2010; Ushijima et al., 2012), the histone methylation may be dynamically regulated in testis. Although few mouse models exist that allow precise detection of methylation activity that directly regulates histone replacement during spermiogenesis, some studies have revealed that histone methylation may modulate the histone-to-protamine transition through some other ways. PYGO2 (Pygopus homolog 2) comprises a C-terminal PHD finger, which can recognize the H3K4me3 and is specifically located in the elongating spermatid nuclei. In mice, the reduction of *Pygo2* influenced the *Tnp, Prm* genes expression and caused the abnormal nuclear condensation, which further led to male sterility (Nair et al., 2008). Furthermore, PYGO2 associates with a histone acetyltransferase (HAT) activity, and the acetylation of H3 is disrupted in *Pygo2* reduced elongating spermatids (Nair et al., 2008), indicating PYGO2 may recognize H3K4me3 through its PHD domain and could recruit HAT to facilitate H3 acetylation and further histone-to-protamine transitions. As described before, PHF7 could recognize the H3K4me3/me2 and catalyze H2A ubiquitination to facilitate the histone-to-protamine transitions (Wang et al., 2019). The predominant histone methyltransferase SETD2 (SET domain–containing 2) catalyzes the H3K36me3, and knocking out *Setd2* in mouse germ cells causes aberrant spermiogenesis, resulting in complete male infertility. Moreover, the disruption of SETD2 causes complete loss of H3K36me3 and impaired activation of *Tnp* and *Prm* genes (Zuo et al., 2018), indicating H3K36me3 may regulate the histone-to-protamine transition by activating *Tnp* and *Prm* genes expression. Contrarily, JHDM2A (JmjC-domain-containing histone demethylase 2A) is an H3K9me2/1-specific demethylase. The loss of *Jhdm2a* in mice exhibits post-meiotic chromatin condensation defects and leads to male infertility. Although global H3K9 methylation has no effect in *Jhdm2a*-null testis, JHDM2A directly binds to and controls H3K9 methylation at the promoter of *Tnp1* and *Prm1* genes, which further regulates the sperm genome packaging and chromatin condensation (Okada et al., 2007).

## Phosphorylation

Histone phosphorylation is involved in various cellular processes (Rossetto et al., 2012; Bao and Bedford, 2016), and dynamic histone phosphorylation have been observed during spermatogenesis (Govin et al., 2010; Bao and Bedford, 2016). The phosphorylation of histone H2AX at residue Ser139 (γH2AX) plays important roles in many biological processes, such as meiotic recombination and male sex chromosome inactivation in germ cells (Li et al., 2005). γH2AX is detectable in elongating spermatids, and TSSK6 has been identified to be responsible for the H2AX phosphorylation during spermiogenesis (Jha et al., 2017). In mice, targeted deletion of *Tssk6* leads to male sterility caused by the impairment in morphology and motility of spermatozoa (Spiridonov et al., 2005). In spermatozoa, the loss of TSSK6 blocks γH2AX formation, resulting in elevated H3, H4 and the precursor and intermediate of PRM2 (Jha et al., 2017). These results indicate that TSSK6 may mediate γH2AX to participate in the histone-to-protamine transition. H4S1 phosphorylation is highly expressed in mouse spermatocyte, round and elongating spermatids (Krishnamoorthy et al., 2006; Zhang et al., 2016). H4S1 phosphorylation has been found to be essential for chromatin compaction and concomitantly histone accessibility (Krishnamoorthy et al., 2006; Wendt and Shilatifard, 2006), suggesting that H4S1 phosphorylation is required for histone replacement during spermiogenesis. Outside the canonical histones, many phosphorylated residues have been identified, using mass spectrometry analyses, that exist on different testis-specific histone variants, such as H1T, HILS1, TH2A, TH2B (Sarg et al., 2009; Pentakota et al., 2014; Mishra et al., 2015; Luense et al., 2016; Hada et al., 2017). Although many core histones and histone variants phosphorylation have been identified in germ cells, their physiological roles need further investigation.

## Other Modifications

A variety of histone lysine modifications have been identified, including butyrylation, crotonylation, malonylation, propionylation, and succinylation (Tan et al., 2011; Sabari et al., 2017). Kcr (Lysine crotonylation) is a newly identified histone modification and is detectable in elongating spermatids, which regulated testis-specific genes activation in post-meiotic germ cells (Tan et al., 2011). The CDYL (chromodomain Y-like) protein, which contains a C-terminal CoAP domain that interacts with CoA to achieve its crotonyltransferase activity, may suppress the histone Kcr by converting crotonyl-CoA to β-hydroxybutyryl-CoA. Accordingly, *Cdyl*-deficient male mice show reduced fertility, decreased epididymal sperm count and sperm cell motility, and dysregulated histone Kcr (Liu et al., 2017b). In the *Cdyl*-deficient mouse testes, further analysis showed that the elevated TP1 and PRM2 were localized in a chromatin-free regions (Liu et al., 2017b), suggesting that histone crotonylation is essential for the histone-to-protamine transition during spermiogenesis.

Poly-ADP-ribosylation (PARsylation) is a common protein PTM (post-translational modification) observed in higher eukaryotes and involved in many different fundamental cellular functions. All of core histones and the linker histone H1 can be ADP-ribosylated (Gagne et al., 2006; Messner and Hottiger, 2011), which could be catalyzed by poly(ADP-ribose) polymerases, such as PARP1 and PARP2, and resolved by PARG (PAR glycohydrolase) (Gibson and Kraus, 2012). The PARP1, PARP2 and PARsylation proteins are specifically detected in elongating spermatids (Meyer-Ficca et al., 2005), and the perturbed PARsylation causes reduced male fertility with abnormal retention of core histones, H1T and HILS1 in mature spermatozoa (Meyer-Ficca et al., 2009; Meyer-Ficca et al., 2011; Meyer-Ficca et al., 2015). Thus, PARsylation is essential for the histone-to-protamine replacement, yet the precise PARsylation histone sites need further characterization.

## Transition Proteins

Between histone eviction and protamine incorporation in the nuclei of spermatids, about ninety percent of the chromatin components consist of TPs, which are arginine- and lysine-rich proteins encoded by *Tnp1* and *Tnp2* (Meistrich et al., 2003). However, the functional roles of each TP are still controversial (Rathke et al., 2014). TP1 could reduce the melting temperature of DNA and relax the DNA from core particles of nucleosome, whereas TP2 tends to compact the nucleosomal DNA by increasing its melting temperature, indicating TP2 may promote DNA condensation while TP1 facilitates the eviction of the histones (Singh and Rao, 1988; Akama et al., 1998; Kolthur-Seetharam et al., 2009; Rathke et al., 2014). However, a separate study that shown that neither TP1 nor TP2 leads to the conformation changes in supercoiled DNA (Levesque et al., 1998). These differences might reveal their unique roles during mammal spermiogenesis, as single knockout of either *Tnp1* or *Tnp2* leads to little morphological alteration of spermatozoa in mouse models. Elevated TP2 and TP1 proteins could be observed in *Tnp1*-null and *Tnp2*-null spermatids, respectively (Yu et al., 2000; Zhao et al., 2001). Thus, TP1 and TP2 may compensate for each other *in vivo.* Indeed, *Tnp1* and *Tnp2* double-knockout mice show severe abnormal spermiogenesis with a general decrease in sperm motility and abnormal sperm morphology (Shirley et al., 2004)*.* The chromatin condensation is perturbed in the *Tnp1* and *Tnp2* double-knockout mice as severe histones retention is detectable, indicating TPs function redundantly yet have unique roles in the histone-to-protamine transition (Shirley et al., 2004; Zhao et al., 2004; Bao and Bedford, 2016).

## Protamines

Protamines are basic proteins that replace TPs in late spermatids (Rathke et al., 2014; Bao and Bedford, 2016). Two protamine genes (*Prm1* and *Prm2*) localize on the same chromosome in both humans and mice (Balhorn, 2007). Protamines tightly interact with DNA *via* a central arginine-rich DNA-binding domain (Balhorn, 2007). Unlike *Tnp* genes, the disruption of either *Prm1* or *Prm2* leads to the male infertility (Cho et al., 2001). Protamines have multiple PTM sites, and a total of 11 PTMs have been identified on the protamines of mouse spermatozoa, including acetylation, phosphorylation and methylation (Brunner et al., 2014). One site of interest is PRM2 S55, which is a candidate phosphorylated substrate residue of CAMK4 (Ca^2+^/calmodulin-dependent protein kinase IV) (Wu et al., 2000). Targeted *Camk4* knockout male mice are infertile, and the transition protein displacement by PRM2 is perturbed as a specific loss of PRM2 and prolonged retention of TP2 in *Camk4*-null spermatids. *In vitro*, PRM2 could be phosphorylated by CAMK4, implicating CAMK4 mediated PRM2 phosphorylation is required for the protamine incorporation during spermiogenesis (Wu et al., 2000). Thus, the specific post-translational modifications on protamines may also be essential for the histone-to-protamine transition.

## Conclusion and Future Perspectives

During the histone-to-protamine transition, many epigenetic regulators work together to facilitate paternal genome re-organization and packaging into the highly condensed nuclei of spermatozoa, through histone variation, specific histone modification and their related chromatin remodelers. Any defects during the histone-to-protamine transition would lead to male infertility (Bao and Bedford, 2016). While the morphological changes during spermiogenesis are well characterized, the precise molecular mechanisms underlying the chromatin re-organization, in particular the transition from histones to protamines, are still unclear. It’s difficult to characterize the dynamic processes that occur during histone eviction, transition protein incorporation and protamine insertion. Moreover, 10% of the spermatozoa population in the epididymis has not yet completed the histone-to-protamine transition (Yoshida et al., 2018). These problems may be ascribed to a lack of experimental methods, which could fully recapitulate germ cell development *in vitro*. Further physiological insights may be gained by developing an *in vitro* germ-cell culture system that more accurately recapitulates the *in vivo* histone-to-protamine transition.

Many histone variants modulate histone replacement by regulating the chromatin structure; therefore, nucleosomes containing these histone variants often maintain a relatively decondensed and open chromatin configuration, facilitating histone replacement during spermiogenesis. The redundant function of histone variants in modulating chromatin configuration ensures that defects in some histone variants have a limited effect on spermatogenesis. Indeed, some mutant histone variants in mouse models are dispensable for male fertility, and mice may show elevated levels of compensatory histones or histone variants. However, the redundant function of histone variants makes it difficult to explore the precise role of each histone variant in histone replacement.

Although many histone modifications have been identified during the histone-to-protamine transition, many studies are descriptive and correlative. The direct manipulation of histone modification sites to reveal function is still urgently needed. With the development of gene editing tools, for example the CRISPR/Cas9 system, mouse models disrupting these histone modifications may be generated and used to elucidate function and *in vivo* relevance in the future. The following open-ended questions still need to be answered to provide in-depth investigation in the field.

In addition to the histone variants and modifications mentioned above, what other novel histone variants and modifications participate in the histone-to-protamine transition? How can we identify them?
How and where do histone variants replace canonical histones? What signal is needed to initiate replacement?
As histone variants and modifications are identified that participate in the histone-to-protamine transition, how do we establish an epigenetic modulating network for this process? Which type of histone code is the initiating code?
Histone hyperacetylation works as a determining event during the histone-to-protamine transition. Is this histone hyperacetylation an initial signal or an indirect consequence of prior events?
What’s the relationship between these histone variants and modifications? What’s the mechanism underlying the cross talk between them?
Chromatin assembly is modulated by histone chaperones or other chromatin remodelers. What’s the functional role of histone chaperones during the histone-to-protamine transition? Which histone variants or modifications send a signal to the chaperones?
(7) How do the transition proteins replace the histone? How do protamines replace the transition proteins? What are the detailed functional roles of transition proteins?
(8) Are there still post-translational modifications that need to be discovered in order to more accurately describe how histone modification plays a role in spermatozoa maturation?

These questions and their underlying ideas need further investigation and refining to help us more thoroughly understand the complex molecular relationships and exact regulating mechanisms of the histone-to-protamine transition.

## Author Contributions

WT and HG wrote the manuscript and drew the figures; CL and WL proposed the idea and revised the manuscript. All authors listed have made a substantial, direct and intellectual contribution to the work and approved it for publication.

## Funding

This work was supported by the Strategic Priority Research Program of the Chinese Academy of Sciences (Grant No. XDA16020701), the National Key R&D Program of China (grant 2016YFA0500901, 2018YFC1004202), the National Natural Science Foundation of China (grants 31771501, 91649202) and the Youth Innovation Promotion Association CAS (2018109).

## Conflict of Interest

The authors declare that the research was conducted in the absence of any commercial or financial relationships that could be construed as a potential conflict of interest.

## References

[B1] Akama K. Sato H. Hasegawa S. Shimada I. Nakano M. (1998). Transition protein 1 from boar late spermatid nuclei having DNA-melting activity is a dimeric protein. Biochem. Mol. Biol. Int. 44, 315–323. 10.1080/15216549800201332 9530514

[B2] Akhtar A. Becker P. B. (2000). Activation of transcription through histone H4 acetylation by MOF, an acetyltransferase essential for dosage compensation in *Drosophila.* Mol. Cell 5, 367–375. 10.1016/S1097-2765(00)80431-1 10882077

[B3] Anuar N. D. Kurscheid S. Field M. Zhang L. Rebar E. Gregory P. (2019). Gene editing of the multi-copy H2A.B gene and its importance for fertility. Genome Biol. 20, 23. 10.1186/s13059-019-1633-3 30704500 PMC6357441

[B4] Awe S. Renkawitz-Pohl R. (2010). Histone H4 acetylation is essential to proceed from a histone- to a protamine-based chromatin structure in spermatid nuclei of *Drosophila melanogaster.* Syst. Biol. in Reprod. Med. 56, 44–61. 10.3109/19396360903490790 20170286

[B5] Baarends W. M. Hoogerbrugge T. W. Roest H. P. Ooms M. Vreeburg J. Hoeijmakers J. H. J. (1999). Histone ubiquitination and chromatin remodeling in mouse spermatogenesis. Dev. Biol. 207, 322–333. 10.1006/dbio.1998.9155 10068466

[B6] Balhorn R. (2007). The protamine family of sperm nuclear proteins. Genome Biol. 8, 1–8. 10.1186/gb-2007-8-9-227 PMC2375014 17903313

[B7] Bao J. Bedford M. T. (2016). Epigenetic regulation of the histone-to-protamine transition during spermiogenesis. Reproduction 151, R55–R70. 10.1530/REP-15-0562 26850883 PMC4896072

[B8] Barral S. Morozumi Y. Tanaka H. Montellier E. Govin J. De Dieuleveult M. (2017). Histone Variant H2A.L.2 Guides transition protein-dependent protamine assembly in male germ cells. Mol. Cell 66, 89–101 e108. 10.1016/j.molcel.2017.02.025 28366643

[B9] Bednar J. Horowitz R. A. Grigoryev S. A. Carruthers L. M. Hansen J. C. Koster A. J. (1998). Nucleosomes, linker DNA, and linker histone form a unique structural motif that directs the higher-order folding and compaction of chromatin. Proc. Natl. Acad. Sci. U. S. A. 95, 14173–14178. 10.1073/pnas.95.24.14173 9826673 PMC24346

[B10] Bell E. L. Nagamori I. Williams E. O. Del Rosario A. M. Bryson B. D. Watson N. (2014). SirT1 is required in the male germ cell for differentiation and fecundity in mice. Development 141, 3495–3504. 10.1242/dev.110627 25142464 PMC4197722

[B11] Berkovits B. D. Wolgemuth D. J. (2013). The role of the double bromodomain-containing BET genes during mammalian spermatogenesis. Gametogenesis 102, 293–326. 10.1016/B978-0-12-416024-8.00011-8 PMC3918955 23287038

[B12] Boskovic A. Torres-Padilla M. E. (2013). How mammals pack their sperm: a variant matter. Genes Dev. 27, 1635–1639. 10.1101/gad.226167.113 23913918 PMC3744721

[B13] Bramlage B. Kosciessa U. Doenecke D. (1997). Differential expression of the murine histone genes H3.3A and H3.3B. Differentiation 62, 13–20. 10.1046/j.1432-0436.1997.6210013.x 9373943

[B14] Brunner A. M. Nanni P. Mansuy I. M. (2014). Epigenetic marking of sperm by post-translational modification of histones and protamines. Epigenet. Chromatin 7, 1–12. 10.1186/1756-8935-7-2 PMC3904194 24443974

[B15] Carmell M. A. Girard A. Van De Kant H. J. G. Bourc’his D. Bestor T. H. De Rooij D. G. (2007). MIWI2 is essential for spermatogenesis and repression of transposons in the mouse male germline. Dev. Cell 12, 503–514. 10.1016/j.devcel.2007.03.001 17395546

[B16] Chen H. Y. Sun J. M. Zhang Y. Davie J. R. Meistrich M. L. (1998). Ubiquitination of histone H3 in elongating spermatids of rat testes. J. Biol. Chem. 273, 13165–13169. 10.1074/jbc.273.21.13165 9582357

[B17] Chen P. Zhao J. C. Wang Y. Wang M. Long H. Z. Liang D. (2013). H3.3 actively marks enhancers and primes gene transcription via opening higher-ordered chromatin. Genes Dev. 27, 2109–2124. 10.1101/gad.222174.113 24065740 PMC3850095

[B18] Cho C. Willis W. D. Goulding E. H. Haesook J. H. Choi Y. C. Hecht N. B. Eddy E. M. (2001). Haploinsufficiency of protamine-1 or-2 causes infertility in mice. Nat. Genet. 28, 82–86. 10.1038/ng0501-82 11326282

[B19] Churikov D. Siino J. Svetlova M. Zhang K. L. Gineitis A. Bradbury E. M. (2004). Novel human testis-specific histone H2B encoded by the interrupted gene on the X chromosome. Genomics 84, 745–756. 10.1016/j.ygeno.2004.06.001 15475252

[B20] Couldrey C. Carlton M. B. L. Nolan P. M. Colledge W. H. Evans M. J. (1999). A retroviral gene trap insertion into the histone 3.3A gene causes partial neonatal lethality, stunted growth, neuromuscular deficits and male sub-fertility in transgenic mice. Hum. Mol. Genet. 8, 2489–2495. 10.1093/hmg/8.13.2489 10556297

[B21] De Vries M. Ramos L. Housein Z. De Boer P. (2012). Chromatin remodelling initiation during human spermiogenesis. Biol. Open 1, 446–457. 10.1242/bio.2012844 23213436 PMC3507207

[B22] Delucia F. Faraonemennella M. R. Derme M. Quesada P. Caiafa P. Farina B. (1994). Histone-induced condensation of rat testis chromatin—testis-specific H1t versus somatic H1 variants. Biochem. Biophys. Res. Commun. 198, 32–39. 10.1006/bbrc.1994.1005 8292037

[B23] Deng W. Lin H. F. (2002). Miwi, a murine homolog of Piwi, encodes a cytoplasmic protein essential for spermatogenesis. Dev. Cell 2, 819–830. 10.1016/S1534-5807(02)00165-X 12062093

[B24] Dhar S. Thota A. Rao M. R. S. (2012). Insights into role of bromodomain, testis-specific (Brdt) in acetylated histone H4-dependent chromatin remodeling in mammalian spermiogenesis. J. Biol. Chem. 287, 6387–6405. 10.1074/jbc.M111.288167 22215678 PMC3307259

[B25] Dong Y. Isono K. Ohbo K. Endo T. A. Ohara O. Maekawa M. (2017). EPC1/TIP60-mediated histone acetylation facilitates spermiogenesis in mice. Mol. Cell. Biol. 37, e00082-17. 10.1128/MCB.00082-17 PMC5599718 28694333

[B26] Dottermusch-Heidel C. Klaus E. S. Gonzalez N. H. Bhushan S. Meinhardt A. Bergmann M. (2014). H3K79 methylation directly precedes the histone-to-protamine transition in mammalian spermatids and is sensitive to bacterial infections. Andrology 2, 655–665. 10.1111/j.2047-2927.2014.00248.x 25079683

[B27] Doyon Y. Selleck W. Lane W. S. Tan S. Cote J. (2004). Structural and functional conservation of the NuA4 histone acetyltransferase complex from yeast to humans. Mol. Cell. Biol. 24, 1884–1896. 10.1128/MCB.24.5.1884-1896.2004 14966270 PMC350560

[B28] Drabent B. Benavente R. Hoyer-Fender S. (2003). Histone H1t is not replaced by H1.1 or H1.2 in pachytene spermatocytes or spermatids of H1t-deficient mice. Cytogenet. Genome Res. 103, 307–313. 10.1159/000076818 15051953

[B29] Drabent B. Bode C. Bramlage B. Doenecke D. (1996). Expression of the mouse testicular histone gene H1t during spermatogenesis. Histochem. Cell Biol. 106, 247–251. 10.1007/BF02484408 8877387

[B30] Drabent B. Saftig P. Bode C. Doenecke D. (2000). Spermatogenesis proceeds normally in mice without linker histone H1t. Histochem. Cell Biol. 113, 433–442. 10933220 10.1007/s004180000146

[B31] Fantz D. A. Hatfield W. R. Horvath G. Kistler M. K. Kistler W. S. (2001). Mice with a targeted disruption of the H1t gene are fertile and undergo normal changes in structural chromosomal proteins during spermiogenesis. Biol. Reprod. 64, 425–431. 10.1095/biolreprod64.2.425 11159343

[B32] Gagne J. P. Hendzel M. J. Droit A. Poirier G. G. (2006). The expanding role of poly(ADP-ribose) metabolism: current challenges and new perspectives. Curr. Opin. Cell Biol. 18, 145–151. 10.1016/j.ceb.2006.02.013 16516457

[B33] Gaucher J. Boussouar F. Montellier E. Curtet S. Buchou T. Bertrand S. (2012). Bromodomain-dependent stage-specific male genome programming by Brdt. EMBO J. 31, 3809–3820. 10.1038/emboj.2012.233 22922464 PMC3463845

[B34] Gibson B. A. Kraus W. L. (2012). New insights into the molecular and cellular functions of poly(ADP-ribose) and PARPs. Nat. Rev. Mol. Cell Biol. 13, 411–424. 10.1038/nrm3376 22713970

[B35] Godmann M. Auger V. Ferraroni-Aguiar V. Di Sauro A. Sette C. Behr R. (2007). Dynamic regulation of histone h3 methylation at lysine 4 in mammalian spermatogenesis. Biol. Reprod. 77, 754–764. 10.1095/biolreprod.107.062265 17634443

[B36] Gou L. T. Kang J. Y. Dai P. Wang X. Li F. Zhao S. (2017). Ubiquitination-deficient mutations in human Piwi cause male infertility by impairing histone-to-protamine exchange during spermiogenesis. Obstet. Gynecol. Survey 72, 540–541. 10.1097/OGX.0000000000000482 PMC5985145 28552346

[B37] Govin J. Caron C. Lestrat C. Rousseaux S. Khochbin S. (2004). The role of histones in chromatin remodelling during mammalian spermiogenesis. Eur. J. Biochem. 271, 3459–3469. 10.1111/j.1432-1033.2004.04266.x 15317581

[B38] Govin J. Dorsey J. Gaucher J. Rousseaux S. Khochbin S. Berger S. L. (2010). Systematic screen reveals new functional dynamics of histones H3 and H4 during gametogenesis. Genes Dev. 24, 1772–1786. 10.1101/gad.1954910 20713519 PMC2922505

[B39] Govin J. Escoffier E. Rousseaux S. Kuhn L. Ferro M. Thevenon J. (2007). Pericentric heterochromatin reprogramming by new histone variants during mouse spermiogenesis. J. Cell Biol. 176, 283–294. 10.1083/jcb.200604141 17261847 PMC2063955

[B40] Govin J. Lestrat C. Caron C. Pivot-Pajot C. Rousseaux S. Khochbin S. (2006). Histone acetylation-mediated chromatin compaction during mouse spermatogenesis. Cancer Ther. 57, 155–15+. 10.1007/3-540-37633-X_9 16568954

[B41] Grimes S. R. Henderson N. (1984a). Acetylation of rat testis histones H2b and Th2b. Dev. Biol. 101, 516–521. 10.1016/0012-1606(84)90165-9 6692994

[B42] Grimes S. R. Jr. Henderson N. (1984b). Hyperacetylation of histone H4 in rat testis spermatids. Exp. Cell Res. 152, 91–97. 10.1016/0014-4827(84)90232-5 6714327

[B43] Hada M. Masuda K. Yamaguchi K. Shirahige K. Okada Y. (2017). Identification of a variant-specific phosphorylation of TH2A during spermiogenesis. Sci. Rep. 7, 1–13. 10.1038/srep46228 28387373 PMC5384234

[B44] Hao S. L. Ni F. D. Yang W. X. (2019). The dynamics and regulation of chromatin remodeling during spermiogenesis. Gene 706, 201–210. 10.1016/j.gene.2019.05.027 31085275

[B45] Happel N. Doenecke D. (2009). Histone H1 and its isoforms: contribution to chromatin structure and function. Gene 431, 1–12. 10.1016/j.gene.2008.11.003 19059319

[B46] Hazzouri M. Pivot-Pajot C. Faure A. K. Usson Y. Pelletier R. Sele B. (2000). Regulated hyperacetylation of core histones during mouse spermatogenesis: involvement of histone-deacetylases. Eur. J. Cell Biol. 79, 950–960. 10.1078/0171-9335-00123 11152286

[B47] Hershko A. Ciechanover A. (1998). The ubiquitin system. Annu. Rev. Biochem. 67, 425–479. 10.1146/annurev.biochem.67.1.425 9759494

[B48] Hess R. A. Renato De Franca L. (2008). Spermatogenesis and cycle of the seminiferous epithelium. Adv. Exp. Med. Biol. 636, 1–15. 10.1007/978-0-387-09597-4_1 19856159

[B49] Hoghoughi N. Barral S. Vargas A. Rousseaux S. Khochbin S. (2018). Histone variants: essential actors in male genome programming. J. Biochem. 163, 97–103. 10.1093/jb/mvx079 29165574

[B50] Hou X. Zhang W. Xiao Z. Gan H. Lin X. Liao S. (2012). Mining and characterization of ubiquitin E3 ligases expressed in the mouse testis. BMC Genomics 13, 495. 10.1186/1471-2164-13-495 22992278 PMC3460789

[B51] Jha K. N. Tripurani S. K. Johnson G. R. (2017). TSSK6 is required for gamma H2AX formation and the histone-to-protamine transition during spermiogenesis. J. Cell Sci. 130, 1835–1844. 10.1242/jcs.202721 28389581

[B52] Juliano C. Wang J. Q. Lin H. F. (2011). Uniting germline and stem cells: the function of Piwi proteins and the piRNA pathway in diverse organisms. Ann. Rev. Genet. 45, 447–469. 10.1146/annurev-genet-110410-132541 21942366 PMC3832951

[B53] Kan P. Y. Caterino T. L. Hayes J. J. (2009). The H4 tail domain participates in intra- and internucleosome interactions with protein and DNA during folding and oligomerization of nucleosome arrays. Mol. Cell. Biol. 29, 538–546. 10.1128/MCB.01343-08 19001093 PMC2612503

[B54] Ketchum C. C. Larsen C. D. Mcneil A. Meyer-Ficca M. L. Meyer R. G. (2018). Early histone H4 acetylation during chromatin remodeling in equine spermatogenesis. Biol. Reprod. 98, 115–129. 10.1093/biolre/iox159 29186293

[B55] Khadake J. R. Rao M. R. S. (1995). DNA-condensing and chromatin-condensing properties of rat testes Hla and hit compared to those of rat-liver Hlbdec—Hlt is a poor condenser of chromatin. Biochemistry 34, 15792–15801. 10.1021/bi00048a025 7495811

[B56] Khor B. Bredemeyer A. L. Huang C. Y. Turnbull I. R. Evans R. Maggi L. B.(2006). Proteasome activator PA200 is required for normal spermatogenesis. Mol. Cell. Biol. 26, 2999–3007. 10.1128/MCB.26.8.2999-3007.2006 16581775 PMC1446934

[B57] Kimura S. Loppin B. (2016). The *Drosophila* chromosomal protein Mst77F is processed to generate an essential component of mature sperm chromatin. Open Biol. 6, 1–12. 10.1098/rsob.160207 PMC5133442 27810970

[B58] Kolthur-Seetharam U. Pradeepa M. M. Gupta N. Narayanaswamy R. Rao M.R.S. (2009). Spatiotemporal organization of AT- and GC-rich DNA and their association with transition proteins TP1 and TP2 in rat condensing spermatids. J. Histochem. Cytochem. 57, 951–962. 10.1369/jhc.2009.953414 19506090 PMC2746728

[B59] Komander D. Rape M. (2012). The ubiquitin code. Ann. Rev. Biochem. 81, 203–229. 10.1146/annurev-biochem-060310-170328 22524316

[B60] Kowalski A. Palyga J. (2012). Linker histone subtypes and their allelic variants. Cell Biol. Int. 36, 981–996. 10.1042/CBI20120133 23075301

[B61] Krishnamoorthy T. Chen X. Govin J. Cheung W. L. Dorsey J. Schindler K. (2006). Phosphorylation of histone H4 Ser1 regulates sporulation in yeast and is conserved in fly and mouse spermatogenesis. Genes Dev. 20, 2580–2592. 10.1101/gad.1457006 16980586 PMC1578680

[B62] Kuramochi-Miyagawa S. Kimura T. Ijiri T. W. Isobe T. Asada N. Fujita Y. (2004). Mili, a mammalian member of piwi family gene, is essential for spermatogenesis. Development 131, 839–849. 10.1242/dev.00973 14736746

[B63] Lee J. Park H. S. Kim H. H. Yun Y. J. Lee D. R. Lee S. (2009). Functional polymorphism in H2BFWT-5'UTR is associated with susceptibility to male infertility. J. Cell. Mol. Med. 13, 1942–1951. 10.1111/j.1582-4934.2009.00830.x 19583817 PMC6529973

[B64] Levesque D. Veilleux S. Caron N. Boissonneault G. (1998). Architectural DNA-binding properties of the spermatidal transition proteins 1 and 2. Biochem. Biophys. Res. Commun. 252, 602–609. 10.1006/bbrc.1998.9687 9837753

[B65] Li A. Eirin-Lopez J. Ausio J. (2005). H2AX: tailoring histone H2A for chromatin-dependent genomic integrity. Biochem. Cell Biol. 83, 505–515. 10.1139/o05-114 16094454

[B66] Liu C. Song Z. H. Wang L. N. Yu H. Y. Liu W. X. Shang Y. L. (2017a). Sirt1 regulates acrosome biogenesis by modulating autophagic flux during spermiogenesis in mice. Development 144, 441–451. 10.1242/dev.147074 28003215

[B67] Liu S. M. Yu H. J. Liu Y. Q. Liu X. H. Zhang Y. Bu C. (2017b). Chromodomain protein CDYL acts as a crotonyl-CoA hydratase to regulate histone crotonylation and spermatogenesis. Mol. Cell 67, 853–85+. 10.1016/j.molcel.2017.07.011 28803779

[B68] Liu Z. L. Zhou S. L. Liao L. Chen X. Meistrich M. Xu J. M. (2010). Jmjd1a Demethylase-regulated histone modification is essential for cAMP-response element modulator-regulated gene expression and spermatogenesis. J. Biol. Chem. 285, 2758–2770. 10.1074/jbc.M109.066845 19910458 PMC2807331

[B69] Lu L. Y. Wu J. X. Ye L. Gavrilina G. B. Saunders T. L. Yu X. C. (2010). RNF8-dependent histone modifications regulate nucleosome removal during spermatogenesis. Dev. Cell 18, 371–384. 10.1016/j.devcel.2010.01.010 20153262 PMC2840054

[B70] Luense L. J. Wang X. S. Schon S. B. Weller A. H. Shiao E. L. Bryant J. M.(2016). Comprehensive analysis of histone post-translational modifications in mouse and human male germ cells. Epigenet. Chromatin 9, 1–15. 10.1186/s13072-016-0072-6 PMC4915177 27330565

[B71] Ma T. Keller J. A. Yu X. C. (2011). RNF8-dependent histone ubiquitination during DNA damage response and spermatogenesis. Acta Biochim. Biophys. Sin. 43, 339–345. 10.1093/abbs/gmr016 21444325 PMC3080603

[B72] Manterola M. Brown T. M. Oh M. Y. Garyn C. Gonzalez B. J. Wolgemuth D. J. (2018). BRDT is an essential epigenetic regulator for proper chromatin organization, silencing of sex chromosomes and crossover formation in male meiosis. PLoS Genet. 14, 1–30. 10.1371/journal.pgen.1007209 PMC5841650 29513658

[B73] Martianov I. Brancorsini S. Catena R. Gansmuller A. Kotaja N. Parvinen M. (2005). Polar nuclear localization of H1T2, a histone H1 variant, required for spermatid elongation and DNA condensation during spermiogenesis. Proc. Natl Acad. Sci. U. S. A. 102, 2808–2813. 10.1073/pnas.0406060102 15710904 PMC549447

[B74] Mccarrey J. R. Geyer C. B. Yoshioka H. (2005). Epigenetic regulation of testis-specific gene expression. Testicular Cell Dyn. Endocr. Signal. 1061, 226–242. 10.1196/annals.1336.025 16467272

[B75] Meistrich M. L. Bucci L. R. Trostleweige P. K. Brock W. A. (1985). Histone variants in rat spermatogonia and primary spermatocytes. Dev. Biol. 112, 230–240. 10.1016/0012-1606(85)90137-X 3932111

[B76] Meistrich M. L. Mohapatra B. Shirley C. R. Zhao M. (2003). Roles of transition nuclear proteins in spermiogenesis. Chromosoma 111, 483–488. 10.1007/s00412-002-0227-z 12743712

[B77] Meng C. Liao J. Zhao D. Huang H. Qin J. Lee T. L. (2019). L3MBTL2 regulates chromatin remodeling during spermatogenesis. Cell Death Differ. 13, 1–14. 10.1038/s41418-019-0283-z PMC6889272 30760872

[B78] Messner S. Hottiger M. O. (2011). Histone ADP-ribosylation in DNA repair, replication and transcription. Trends Cell Biol. 21, 534–542. 10.1016/j.tcb.2011.06.001 21741840

[B79] Meyer-Ficca M. L. Ihara M. Bader J. J. Leu N. A. Beneke S. Meyer R. G. (2015). Spermatid head elongation with normal nuclear shaping requires ADP-ribosyltransferase PARP11 (ARTD11) in Mice. Biol. Reprod. 92, 1–13. 10.1095/biolreprod.114.123661 PMC4376083 25673562

[B80] Meyer-Ficca M. L. Ihara M. Lonchar J. D. Meistrich M. L. Austin C. A. Min W. (2011). Poly(ADP-ribose) metabolism is essential for proper nucleoprotein exchange during mouse spermiogenesis. Biol. Reprod. 84, 218–228. 10.1095/biolreprod.110.087361 20881315 PMC3071264

[B81] Meyer-Ficca M. L. Lonchar J. Credidio C. Ihara M. Li Y. Wang Z. Q.(2009). Disruption of poly(ADP-ribose) homeostasis affects spermiogenesis and sperm chromatin integrity in mice. Biol. Reprod. 81, 46–55. 10.1095/biolreprod.108.075390 19264700 PMC3110478

[B82] Meyer-Ficca M. L. Scherthan H. Burkle A. Meyer R. G. (2005). Poly(ADP-ribosyl)ation during chromatin remodeling steps in rat spermiogenesis. Chromosoma 114, 67–74. 10.1007/s00412-005-0344-6 15838619

[B83] Mishra L. N. Gupta N. Rao S. M. R. (2015). Mapping of post-translational modifications of spermatid-specific linker histone H1-like protein, HILS1. J. Proteomics 128, 218–230. 10.1016/j.jprot.2015.08.001 26257145

[B84] Mishra L. N. Shalini V. Gupta N. Ghosh K. Suthar N. Bhaduri U.(2018). Spermatid-specific linker histone HILS1 is a poor condenser of DNA and chromatin and preferentially associates with LINE-1 elements. Epigenet. Chromatin 11, 1–21. 10.1186/s13072-018-0214-0 PMC6069787 30068355

[B85] Montellier E. Boussouar F. Rousseaux S. Zhang K. Buchou T. Fenaille F. (2013). Chromatin-to-nucleoprotamine transition is controlled by the histone H2B variant TH2B. Genes Dev. 27, 1680–1692. 10.1101/gad.220095.113 23884607 PMC3744726

[B86] Moss S. B. Orth J. M. (1993). Localization of a spermatid-specific histone-2b protein in mouse spermiogenic cells. Biol. Reprod. 48, 1047–1056. 10.1095/biolreprod48.5.1047 8481469

[B87] Nair M. Nagamori I. Sun P. Mishra D. P. Rheaume C. Li B. (2008). Nuclear regulator Pygo2 controls spermiogenesis and histone H3 acetylation. Dev. Biol. 320, 446–455. 10.1016/j.ydbio.2008.05.553 18614164 PMC2553271

[B88] Okada Y. Scott G. Ray M. K. Mishina Y. Zhang Y. (2007). Histone demethylase JHDM2A is critical for Tnp1 and Prm1 transcription and spermatogenesis. Nature 450, 119–11+. 10.1038/nature06236 17943087

[B89] Oliva R. Bazett-Jones D. Mezquita C. Dixon G. H. (1987). Factors affecting nucleosome disassembly by protamines *in vitro.* Histone hyperacetylation and chromatin structure, time dependence, and the size of the sperm nuclear proteins. J. Biol. Chem. 262, 17016–17025. 3680288

[B90] Oliva R. Mezquita C. (1986). Marked differences in the ability of distinct protamines to disassemble nucleosomal core particles in vitro. Biochemistry 25, 6508–6511. 10.1021/bi00369a025 3790536

[B91] Padavattan S. Shinagawa T. Hasegawa K. Kumasaka T. Ishii S. Kumarevel T. (2015). Structural and functional analyses of nucleosome complexes with mouse histone variants TH2a and TH2b, involved in reprogramming. Biochem. Biophys. Res. Commun. 464, 929–935. 10.1016/j.bbrc.2015.07.070 26188507

[B92] Padavattan S. Thiruselvam V. Shinagawa T. Hasegawa K. Kumasaka T. Ishii S. (2017). Structural analyses of the nucleosome complexes with human testis-specific histone variants, hTh2a and hTh2b. Biophys. Chem. 221, 41–48. 10.1016/j.bpc.2016.11.013 27992841

[B93] Pentakota S. K. Sandhya S. Sikarwar A. P. Chandra N. Rao M. R. S. (2014). Mapping post-translational modifications of mammalian testicular specific histone variant TH2B in tetraploid and haploid germ cells and their implications on the dynamics of nucleosome structure. J. Proteome Res. 13, 5603–5617. 10.1021/pr500597a 25252820

[B94] Pickart C. M. (2001). Mechanisms underlying ubiquitination. Annu. Rev. Biochem. 70, 503–533. 10.1146/annurev.biochem.70.1.503 11395416

[B95] Pivot-Pajot C. Caron C. Govin M. Vion A. Rousseaux S. Khochbin S. (2003). Acetylation-dependent chromatin reorganization by BRDT, a testis-specific bromodomain-containing protein. Mol. Cell. Biol. 23, 5354–5365. 10.1128/MCB.23.15.5354-5365.2003 12861021 PMC165724

[B96] Qian M. X. Pang Y. Liu C. H. Haratake K. Du B. Y. Ji D. Y. (2013). Acetylation-mediated proteasomal degradation of core histones during DNA repair and spermatogenesis. Cell 153, 1012–1024. 10.1016/j.cell.2013.04.032 23706739 PMC3983474

[B97] Rafatmanesh A. Nikzad H. Ebrahimi A. Karimian M. Zamani T. (2018). Association of the c.-9C > T and c.368A > G transitions in H2BFWT gene with male infertility in an Iranian population. Andrologia 50, 1–6. 10.1111/and.12805 28370107

[B98] Raja S. J. Renkawitz-Pohl R. (2005). Replacement by *Drosophila melanogaster* protamines and Mst77F of histones during chromatin condensation in late spermatids and role of sesame in the removal of these proteins from the male pronucleus. Mol. Cell. Biol. 25, 6165–6177. 10.1128/MCB.25.14.6165-6177.2005 15988027 PMC1168805

[B99] Rathke C. Baarends W. M. Awe S. Renkawitz-Pohl R. (2014). Chromatin dynamics during spermiogenesis. Biochim. Biophys. Acta 1839, 155–168. 10.1016/j.bbagrm.2013.08.004 24091090

[B100] Roosen-Runge E. C. (1962). The process of spermatogenesis in mammals. Biol. Rev. Camb Philos. Soc. 37, 343–377. 10.1111/j.1469-185X.1962.tb01616.x 14493721

[B101] Rossetto D. Avvakumov N. Cote J. (2012). Histone phosphorylation A chromatin modification involved in diverse nuclear events. Epigenetics 7, 1098–1108. 10.4161/epi.21975 22948226 PMC3469451

[B102] Sabari B. R. Zhang D. Allis C. D. Zhao Y. M. (2017). Metabolic regulation of gene expression through histone acylations. Nat. Rev. Mol. Cell Biol. 18, 90–101. 10.1038/nrm.2016.140 27924077 PMC5320945

[B103] Sarg B. Chwatal S. Talasz H. Lindner H. H. (2009). Testis-specific linker histone H1t is multiply phosphorylated during spermatogenesis identification of phosphorylation siTES. J. Biol. Chem. 284, 3610–3618. 10.1074/jbc.M805925200 19043117

[B104] Shang E. Y. Nickerson H. D. Wen D. C. Wang X. Y. Wolgemuth D. J. (2007). The first bromodomain of Brdt, a testis-specific member of the BET sub-family of double-bromodomain-containing proteins, is essential for male germ cell differentiation. Development 134, 3507–3515. 10.1242/dev.004481 17728347

[B105] Shinagawa T. Huynh L. M. Takagi T. Tsukamoto D. Tomaru C. Kwak H. G. (2015). Disruption of Th2a and Th2b genes causes defects in spermatogenesis. Development 142, 1287–1292. 10.1242/dev.121830 25742800

[B106] Shiraishi K. Shindo A. Harada A. Ohkawa Y. Kurumizaka H. Kimura H.(2017). Roles of histone H3.5 in human spermatogenesis and spermatogenic disorders. J. Urol. 197, E85–E85. 10.1016/j.juro.2017.02.277 29179259

[B107] Shires A. Carpenter M. P. Chalkley R. (1975). New histones found in mature mammalian testes. Proc. Natl. Acad. Sci. U. S. A. 72, 2714–2718. 10.1073/pnas.72.7.2714 170615 PMC432841

[B108] Shires A. Carpenter M. P. Chalkley R. (1976). Cysteine-containing H2b-like histone found in mature mammalian testis. J. Biol. Chem. 251, 4155–4158. 932025

[B109] Shirley C. R. Hayashi S. Mounsey S. Yanagimachi R. Meistrich M. L. (2004). Abnormalities and reduced reproductive potential of sperm from Tnp1- and Tnp2-null double mutant mice. Biol. Reprod. 71, 1220–1229. 10.1095/biolreprod.104.029363 15189834

[B110] Shogren-Knaak M. Ishii H. Sun J. M. Pazin M. J. Davie J. R. Peterson C. L. (2006). Histone H4-K16 acetylation controls chromatin structure and protein interactions. Science 311, 844–847. 10.1126/science.1124000 16469925

[B111] Singh J. Rao M. R. S. (1988). Interaction of rat testis protein, Tp, with nucleosome core particle. Biochem. Int. 17, 701–710. 3240317

[B112] Siomi M. C. Sato K. Pezic D. Aravin A. A. (2011). PIWI-interacting small RNAs: the vanguard of genome defence. Nat. Rev. Mol. Cell Biol. 12, 246–258. 10.1038/nrm3089 21427766

[B113] Soboleva T. A. Nekrasov M. Pahwa A. Williams R. Huttley G. A. Tremethick D. J. (2012). A unique H2A histone variant occupies the transcriptional start site of active genes. Nat. Struct. Mol. Biol. 19, 25–U37. 10.1038/nsmb.2161 22139013

[B114] Soboleva T. A. Parker B. J. Nekrasov M. Hart-Smith G. Tay Y. J. Tng W. Q. (2017). A new link between transcriptional initiation and pre-mRNA splicing: the RNA binding histone variant H2A.B. PloS Genet. 13, 1–31. 10.1371/journal.pgen.1006633 PMC5345878 28234895

[B115] Song N. Liu J. An S. C. Nishino T. Hishikawa Y. Koji T. (2011). Immunohistochemical analysis of histone H3 modifications in germ cells during mouse spermatogenesis. Acta Histochem. Cytochem. 44, 183–190. 10.1267/ahc.11027 21927517 PMC3168764

[B116] Spiridonov N. A. Wong L. Zerfas P. M. Starost M. F. Pack S. D. Paweletz C. P. (2005). Identification and characterization of SSTK, a serine/threonine protein kinase essential for male fertility. Mol. Cell. Biol. 25, 4250–4261. 10.1128/MCB.25.10.4250-4261.2005 15870294 PMC1087724

[B117] Stadtmueller B. M. Hill C. P. (2011). Proteasome Activators. Mol. Cell 41, 8–19. 10.1016/j.molcel.2010.12.020 21211719 PMC3040445

[B118] Tachiwana H. Kagawa W. Osakabe A. Kawaguchi K. Shiga T. Hayashi-Takanaka Y. (2010). Structural basis of instability of the nucleosome containing a testis-specific histone variant, human H3T. Proc. Natl. Acad. Sci. U. S. A. 107, 10454–10459. 10.1073/pnas.1003064107 20498094 PMC2890842

[B119] Talbert P. B. Henikoff S. (2010). Histone variants - ancient wrap artists of the epigenome. Nat. Rev. Mol. Cell Biol. 11, 264–275. 10.1038/nrm2861 20197778

[B120] Tan M. J. Luo H. Lee S. Jin F. L. Yang J. S. Montellier E. (2011). Identification of 67 histone marks and histone lysine crotonylation as a new type of histone modification. Cell 146, 1015–1027. 10.1016/j.cell.2011.08.008 PMC3176443 21925322

[B121] Tanaka H. Iguchi N. Isotani A. Kitamura K. Toyama Y. Matsuoka Y. (2005). HANP1/H1T2, a novel histone H1-like protein involved in nuclear formation and sperm fertility. Mol. Cell. Biol. 25, 7107–7119. 10.1128/MCB.25.16.7107-7119.2005 16055721 PMC1190238

[B122] Tang M. C. W. Jacobs S. A. Mattiske D. M. Soh Y. M. Graham A. N. Tran A. (2015). Contribution of the two genes encoding histone variant H3.3 to viability and fertility in mice. PloS Genet. 11, 1–23. 10.1371/journal.pgen.1004964 PMC4335506 25675407

[B123] Teimouri M. Najaran H. Hosseinzadeh A. Mazoochi T. (2018). Association between two common transitions of H2BFWT gene and male infertility: a case-control, meta, and structural analysis. Andrology 6, 306–316. 10.1111/andr.12464 29453813

[B124] Thakar A. Gupta P. Ishibashi T. Finn R. Silva-Moreno B. Uchiyama S. Fukui (2009). H2A.Z and H3.3 histone variants affect nucleosome structure: biochemical and biophysical studies. Biochemistry 48, 10852–10857. 10.1021/bi901129e 19856965

[B125] Trostleweige P. K. Meistrich M. L. Brock W. A. Nishioka K. Bremer J. W. (1982). Isolation and characterization of Th2a, a germ cell-specific variant of histone-2a in rat testis. J. Biol. Chem. 257, 5560–5567. 7068607

[B126] Tse C. Sera T. Wolffe A. P. Hansen J. C. (1998). Disruption of higher-order folding by core histone acetylation dramatically enhances transcription of nucleosomal arrays by RNA polymerase III. Mol. Cell. Biol. 18, 4629–4638. 10.1128/MCB.18.8.4629 9671473 PMC109049

[B127] Ueda J. Harada A. Urahama T. Machida S. Maehara K. Hada M.(2017). Testis-specific histone variant H3t gene is essential for entry into spermatogenesis. Cell Rep. 18, 593–600. 10.1016/j.celrep.2016.12.065 28099840

[B128] Unni E. Zhang Y. Kangasniemi M. Saperstein W. Moss S. B. Meistrich M. L. (1995). Stage-specific distribution of the spermatid-specific histone 2b in the rat testis. Biol. Reprod. 53, 820–826. 10.1095/biolreprod53.4.820 8547476

[B129] Urahama T. Harada A. Maehara K. Horikoshi N. Sato K. Sato Y.(2016). Histone H3.5 forms an unstable nucleosome and accumulates around transcription start sites in human testis. Epigenet. Chromatin 9, 1–16. 10.1186/s13072-016-0051-y PMC4714512 26779285

[B130] Urahama T. Horikoshi N. Osakabe A. Tachiwana H. Kurumizaka H. (2014). Structure of human nucleosome containing the testis-specific histone variant TSH2B. Acta Crystallogr. Sect. F-Struct. Biol. Commun. 70, 444–449. 10.1107/S2053230X14004695 24699735 PMC3976059

[B131] Ushijima Y. Inoue Y. H. Konishi T. Kitazawa D. Yoshida H. Shimaji K. (2012). Roles of histone H3K9 methyltransferases during *Drosophila* spermatogenesis. Chrom. Res. 20, 319–331. 10.1007/s10577-012-9276-1 22476432

[B132] Ustrell V. Pratt G. Gorbea C. Rechsteiner M. (2005). Purification and assay of proteasome activator PA200. Methods Enzymol. 398, 321–329. 10.1016/S0076-6879(05)98026-9 16275339

[B133] Van Der Heijden G. W. Derijck A. a. H. A. Posfai E. Giele M. Pelczar P. Ramos L. (2007). Chromosome-wide nucleosome replacement and H3.3 incorporation during mammalian meiotic sex chromosome inactivation. Nat. Genet. 39, 251–258. 10.1038/ng1949 17237782

[B134] Wang X. Kang J. Y. Wei L. Yang X. Sun H. Yang S. (2019). PHF7 is a novel histone H2A E3 ligase prior to histone-to-protamine exchange during spermiogenesis. Development 146, 1–11. 10.1242/dev.175547 31189663

[B135] Welchman R. L. Gordon C. Mayer R. J. (2005). Ubiquitin and ubiquitin-like proteins as multifunctional signals. Nat. Rev. Mol. Cell. Biol. 6, 599–609. 10.1038/nrm1700 16064136

[B136] Wendt K. D. Shilatifard A. (2006). Packing for the germy: the role of histone H4 Ser1 phosphorylation in chromatin compaction and germ cell development. Genes Dev. 20, 2487–2491. 10.1101/gad.1477706 16980578

[B137] Wu J. Y. Ribar T. J. Cummings D. E. Burton K. A. Mcknight G. S. Means A. R. (2000). Spermiogenesis and exchange of basic nuclear proteins are impaired in male germ cells lacking Camk4. Nat. Genet. 25, 448–452. 10.1038/78153 10932193

[B138] Yan W. Ma L. Burns K. H. Matzuk M. M. (2003). HILS1 is a spermatid-specific linker histone H1-like protein implicated in chromatin remodeling during mammalian spermiogenesis. Proc. Natl. Acad. Sci. U. S. A. 100, 10546–10551. 10.1073/pnas.1837812100 12920187 PMC193598

[B139] Ying H. Q. Scott M. B. Zhou-Cun A. (2012). Relationship of SNP of H2BFWT gene to male infertility in a Chinese population with idiopathic spermatogenesis impairment. Biomarkers 17, 402–406. 10.3109/1354750X.2012.677066 22509975

[B140] Yoshida K. Muratani M. Araki H. Miura F. Suzuki T. Dohmae N. (2018). Mapping of histone-binding sites in histone replacement-completed spermatozoa. Nat. Commun. 9, 1–11. 10.1038/s41467-018-06243-9 30250204 PMC6155156

[B141] Yu Y. E. Zhang Y. Unni E. Shirley C. R. Deng J. M. Russell L. D. (2000). Abnormal spermatogenesis and reduced fertility in transition nuclear protein 1-deficient mice. Proc. Natl. Acad. Sci. U. S. A. 97, 4683–4688. 10.1073/pnas.97.9.4683 10781074 PMC18293

[B142] Yuen B.T.K. Bush K. M. Barrilleaux B. L. Cotterman R. Knoepfler P. S. (2014). Histone H3.3 regulates dynamic chromatin states during spermatogenesis. Development 141, 3483–3494. 10.1242/dev.106450 25142466 PMC4197731

[B143] Zhang Z. H. Mu S. M. Guo M. S. Wu J. L. Li Y. Q. Zhang H. (2016). Dynamics of histone H2A, H4 and HS1ph during spermatogenesis with a focus on chromatin condensation and maturity of spermatozoa. Sci. Rep. 6, 1–11. 10.1038/srep25089 27121047 PMC4848542

[B144] Zhao M. Shirley C. R. Mounsey S. Meistrich M. L. (2004). Nucleoprotein transitions during spermiogenesis in mice with transition nuclear protein Tnp1 and Tnp2 mutations. Biol. Reprod. 71, 1016–1025. 10.1095/biolreprod.104.028191 15163613

[B145] Zhao M. Shirley C. R. Yu Y. E. Mohapatra B. Zhang Y. Unni E. (2001). Targeted disruption of the transition protein 2 gene affects sperm chromatin structure and reduces fertility in mice. Mol. Cell Biol. 21, 7243–7255. 10.1128/MCB.21.21.7243-7255.2001 11585907 PMC99899

[B146] Zuo X. L. Rong B. W. Li L. Lv R. T. Lan F. Tong M. H. (2018). The histone methyltransferase SETD2 is required for expression of acrosin-binding protein 1 and protamines and essential for spermiogenesis in mice. J. Biol. Chem. 293, 9188–9197. 10.1074/jbc.RA118.002851 29716999 PMC6005419

